# Supplementary material for: Clinical validation of the short and long UNESP-Botucatu scales for feline pain assessment
Source: PeerJ. 2021 Apr 12;9:e11225. doi: 10.7717/peerj.11225 (PMC8048399; doi:10.7717/peerj.11225)
Supplement: Supplemental Information 3 [file peerj-09-11225-s003.doc]

**
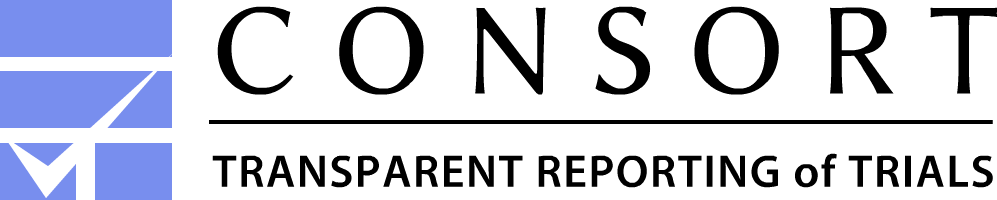
**

**CONSORT 2010 Flow Diagram**

**Allocation**

**Analysis**

**Follow-Up**

**Enrollment**

Assessed for eligibility (n = 53)

Control group (pain free) (n = 10)

Analysed (n = 20)
♦ Excluded from analysis (n = 0)

Analysed – only responsiveness to rescue analgesia (n = 16)
♦ Excluded from responsiveness to rescue analgesia analysis (cats that not required the administration of rescue analgesia) (n = 4)

Lost to follow-up (n = 0)

Discontinued intervention (n = 0)

Allocated to intervention – Pain assessment post surgery (n = 20)

♦ Received allocated intervention (n = 20)

♦ Did not receive allocated intervention (n = 0)

Lost to follow-up (n = 0)

Discontinued intervention (n =0)

Allocated to intervention – Pain assessment in medical care (n = 20)

♦ Received allocated intervention (n=20)

♦ Did not receive allocated intervention (n = 0)

Analysed (n = 20)
♦ Excluded from analysis (n = 0)

Analysed – only responsiveness to rescue analgesia (n = 13)
♦ Excluded from responsiveness to rescue analgesia analysis (cats that required sedation) (n = 7)

Recruited (n = 40)

Excluded (n = 3)

♦  Post-surgery intensive care (n = 2)

♦  Aggressive Behavior (n = 1)
